# A comparison of chyme reinfusion therapy methods: Two case studies

**DOI:** 10.1016/j.intf.2025.100050

**Published:** 2025-03-29

**Authors:** W.Tehana.S. Perera, Hardy Gil, Ishan De Zoysa

**Affiliations:** a National Hospital Sri Lanka, Colombo, Sri Lanka; b Ipanema Research Trust, Auckland, New Zealand

**Keywords:** Chyme reinfusion, Nutritional management, Intestinal failure

## Abstract

**Background:**

High output enterostomy associated malnutrition is a feature of intestinal failure (IF). Chyme reinfusion therapy (CRT) is recommended for restoration of physiological digestive processes in type 2 IF patients. Nevertheless, widespread use of manual CRT has limitations. Recently, innovative technology has demonstrated enhanced intestinal absorption and decreased intestinal losses, associated with improved nutritional and clinical outcomes.

**Case Report:**

A pilot comparison of pump-assisted versus manual CRT was conducted on two short bowel syndrome (SBS) cases using a check list and key informant interviews.

**Conclusion:**

Manual CRT was unpopular among nurses and caregivers, causing frequent interruptions in the procedure. Alternatively, pump-assisted CRT was safe, well tolerated by the patients and accepted by caregivers. The automated system has physiological advantages over parenteral nutrition (PN) with relatively few complications. It could be a cost effective and valuable addition to IF nutritional management but requires verification in a larger planned comparative study.

## Introduction

Small bowel obstruction, inflammation, and fistulas often necessitate double barrel enterostomies, typically reversed after a few months once the acute clinical conditions have stabilized, and patients are nutritionally repleted. However, enterostomies can lead to complications, including dehydration, electrolyte imbalances, renal failure, micronutrient deficiencies, and malnutrition that contribute to hospital re-admissions, increased healthcare costs, and decreased quality of life (QOL) [1]. Patients with high output enterostomies are at risk of intestinal failure (IF) from short bowel syndrome (SBS) when a significant portion of the small intestine is removed or rendered non-functional, resulting in malabsorption and nutrient deficiencies, that need nutritional management. Adults with less than one-third of the jejuno-ileal segment (≤200 cm) typically experience SBS often requiring parenteral nutrition (PN) or intravenous support, depending on their oral intake and the absorptive capacity of the remaining bowel [2]. Within two years, the remaining intestine usually adapts with improved nutrient absorption facilitated by enteral nutrition (EN) [3], [4].

Chyme reinfusion therapy (CRT) collects partially digested food (chyme) from the afferent limb of the small bowel and reintroduces it into the efferent distal limb of the digestive tract, in patients with high-output stomas. CRT re-establishes small bowel continuity, helps maintain intestinal function, aids nutrient absorption with improved nutritional status, and by promoting gut adaptation allows for the early discontinuation of PN, thereby minimizing PN-associated complications [1], [5], [8], [9]

Traditionally manual CRT has been standard practice in Sri Lanka. This requires meticulous handling and depends heavily on the skill and experience of healthcare professionals. A common complication is tube blockage, requiring the effluent to be strained before reinfusion, making the process labor-intensive, time consuming and unpleasant, posing challenges to continuity of care. Recent technological developments automate the chyme collection and reinfusion process [6]. Pump-assisted closed systems, standardize the CRT procedure, minimize human error, with reduced workload for healthcare providers, potentially improving patient comfort and outcomes [7]. However, there is a lack of comparative studies evaluating the relative efficacy, safety, and outcomes of the two methods. Understanding these differences is crucial for optimizing patient care, improving clinical protocols, and guiding future CRT research.

## Case reports

### Objectives

Compare safety, efficacy, cost, and ease of use for manual and pump-assisted CRT

**Aim** To conduct a comparison of manual and pump-assisted CRT that will offer valuable insights for future research to guide clinical practice in Sri Lanka.

### Methodology

Study Design: Comparative, observational Case study on two high stomal output SBS patients

Inclusion Criteria: Patients admitted to surgical units who underwent small bowel resection, ending up with SBS and high output stomas.

Exclusion criteria: Patients who were not able to give their consent. (Altered conscious level, Age < 16 years)

## Results

Quantitative data collection and analysis: Patient parameters were collected by the principal investigator (WTSP) according to a check list (summarized in Table 1) that contrasts the two techniques across key outcome measures: efficacy, safety, cost, and patient outcome, to highlight the strengths and weaknesses of each method based on the collected data.

**Table 1:** **Summary Checklist,** A checklist was completed for each patient by the principal investigator. The checklist covered epidemiological data, procedure, cost, effectiveness, patient’s outcome, and satisfaction. Data were then summarized into Table 1**.**

**Component**	**Manual CRT**	**Pump-assisted CRT**
**Patient Information**		
Patient identification (Bed Head Ticket Number)	179700/2023	77508/2024
Age	17 years	58 years
Gender	Female	Male
Initial Diagnosis and presentation	? Intestinal tuberculosis (TB)with Ileal perforation	Descending colon tumor, causing intestinal obstruction, small bowel distention and ileal tear
Reason for CRT	High output Ileostomy	High output Ileostomy
Anticipated follow up Surgery	Ileo-colic anastomosis	Ileostomy reversal and Hartman’s procedure
**Procedure details**		
Duration of CRT	5 weeks (Patient died)	11 weeks
Complications during the CRT procedure	Abdominal pain, discomfort	No significant complication
Complications after the CRT procedure	Abdominal pain, discomfort	No significant complication
**Efficacy of the procedure**		
Volume reinfused	700 ml/d (50 % of total output)	Almost all the content (Nearly 100 % of output)
Duration for a CRT session	Approximately 2 hours	5 minutes
Completeness of reinfusion	Incomplete (Particles were strained out)	Complete
Frequency of the procedure	Infrequent	Frequent
**Cost**		
Extra staff hours	Needed	Not needed
Supplementary nutrition	Needed. Supplementary PN and ONS	ONS only
Other consumables	Needed (wide bore catheters, 50 cc Syringes, strainers, containers purchased from outside the hospital)	Not needed, all were included in the product package
**Patient Outcome**		
Initial Anthropometry	Weight:28Kg Height:1.43 m BMI: 13.7Kgm^−2^	Weight:44 kg Height:1.68 m BMI:15.5 Kgm^−2^
Weight change/gain	Minimal	3 Kg weight gain over 8 weeks CRT
BMI Change	Minimal, (WHO BMI/Age chart <−2SD)	Improved
Muscle mass/Strength	Minimal/low hand Grip Strength: 11 Kg	Hand Grip strength improved. (32→ 38.5 Kg)
Micronutrient deficiencies	Significant deficiencies of Vit D, B complex	Not significant
Hydration	Features of some dehydration	Not significant
Time taken for stomal reversal	Impossible	Possible after 11 weeks
Serum Albumin	1.9 g/dl→ 2.6 g/dl	3 g/dl → 3.3 g/dl
Serum Na/K	Normal range with fluctuations 139/3.9 mmol/L	Normal range 135/4.7 mmol/L
ALT, AST	127 U/L, 26 U/L	30 U/L, 30 U/L
**Patient’s Quality of Life**		
Mobilization	To chair	Freely (in the ward)
Dependency on Basic Activities of Daily Life	Totally dependent	Totally independent
Improvement in appetite and food intake	None	Significantly
Discomfort while on the procedure	Yes	No
**Patient follow-up**		
Outcome	Gradual deterioration of her condition. Expired	Underwent reconstructive surgery successfully after transferring back to the referral hospital
Follow-up schedule	N/A	Followed up at clinic, initially 2 weekly then monthly.

Qualitative data: Staff /Caregiver experiences were collected by interviews conducted by the principal investigator using the informant’s preferable language. Responses from these interviews were analyzed and the feedback categorized into the themes of; efficacy, safety, cost, ease of use, comfort, overall satisfaction, QOL and clinical outcomes (summarized in Table 2)

Case 1 17-year-old severely malnourished female from a general surgical unit presented with ileal perforation. A double barrel ileostomy leading to SBS was created following resection of the jejunum and proximal ileum. Despite nasogastric (NG) feeds and medical management with loperamide 12 mg/d, daily input/output charts recorded high ileostomy output (1.2–1.5 L). Manual CRT for 5 weeks through a NG tube via distal loop, was difficult and infrequent but decreased output to 0.6 L/d. However, supplementary PN was needed to meet the energy gap,

Case 2 56-year-old male with descending colon tumour, presented with intestinal obstruction and distended small bowel loops with an ileal tear. Surgical resection of the diseased small intestine, with creation of double-barrelled ileostomy was performed, resulting in SBS. For pump assisted CRT, a special feeding tube was inserted into the downstream limb and connected to a small centrifugal pump within the stoma bag. An external electronic driver coupled magnetically to drive the pump and reinfuse the chyme regularly from the bag, reducing output from 1.75 L to 0.36 L within 3 days.

**Table 2:** **Analysis of key informant interviews,** Key informant interviews were conducted by the principal investigator. Data was categorized into four broad themes: **Procedure, Efficacy, Cost, Outcome**. Under each theme several factors were identified. Data were analyzed and summarized in Table 2 below.

**Theme 1: Procedure**		
**Codes**	**Manual CRT**	**Pump-assisted CRT**
1.1. Technique	Initial introduction of the procedure by a senior doctor in the ward *(Senior Registrar)* Thereafter periodical supervision by the House officer necessary. (*Doctor−1)* Bystander couldn’t do the procedure by herself, needed nurses’ assistance *(Bystander)*	Initial demonstration of the machine by a senior House Officer by referring to the instruction leaflet. Thereafter the procedure was carried out by the nurses (no supervision by a doctor) who handed over the machine for the patient to manage the procedure by himself (*Nurse−2*)
1.2 Feeds	NG feeds with blenderised food and ONS(*Doctor−1*)	Normal Oral diet (*Doctor−2*)
1.3 Parenteral Nutrition (PN)	Needed. Patient not able to maintain a central line. PN given via peripheral line but peripheral cannulations difficult (*Doctor−1*)	PN not needed (*Doctor−2*)
1.4 Tubes for the Distal limb	Not available, large bore urinary catheter was used. (*Doctor−1*)	Not needed. The pump-assisted equipment set had the distal tube. (*Nurse −2*)
1.5 Advantages encountered	*Able to use the distal intestine function. (*Doctor−1*)	*Able to use the rest of the intestine (*Doctor−2*) *No need to manually handle the stomal bag contents (*Patient−2*) *Minimal discomfort for the patient (Patient−2) *No spilling of the stomal content to the surrounding (*Patient*−2) *Improved appetite and food intake (*Patient*−2)
1.6 Disadvantages encountered	*Reflux of the reinfused content from the distal limb (*Bystander*) *Needed to clean the clothes and bed linen frequently due to leaking of the content while handling manually. (*Bystander*) *It was an unpleasant experience to handle partially formed stools manually (*Bystander*) *Patient distressed due to the procedure (*Bystander*) *Mother and the staff got exhausted following the procedure (*Nurse−1*) *Frequency and the reinfusion volume will be varied (*Doctor−1*)	*Chance of displacement of the distal tube to outside (*Nurse−2 & Patient-2*) *Need to replace the magnetic pump frequently (*Nurse−2 & Patient −2)*
1.7 Complications	*During the procedure- Abdominal pain and discomfort (*Bystander*) *After the procedure-Pain and discomfort (*Nurse−1)*	Nothing significant (*Patient−2)*
**Theme 2: Efficacy**		
2.1 Frequency	Couldn’t manage in expected frequency. Sometimes the stomal content was discarded when nurses were busy with other work (*Bystander*)	Done regularly by the patient, when the stomal bag was 2/3rd filled. (*Patient*)
2.2 Volume reinfused	Major part was discarded including the particles. Only the liquid part was reinfused. (*Bystander*)	Whole content including the particles were reinfused. (*Patient)*
2.3 Duration of the procedure	Nearly 2 hours (*Nurse −1)*	Roughly 5 minutes (*Nurse−2 & Patient)*
2.4 Overall comment	“Gained very little after hours of struggle” (*Nurse −1)*	“Very effective” (*Nurse−2 & Patient)*
**Theme 3. Cost**		
3.1 Equipment	Needed to obtain from outside the hospital by the caregivers. (*Nurse −1*)	All necessary equipment was available in the pack. (*Nurse−2 & Patient*) Initial pump equipment cost may be high (*Doctor−2)*
3.2 Extra Nursing hours	Extra nursing duty was allocated separately for this patient (*Nurse−1*)	Not needed. Routinely allocated nurse was contacted when there was an issue. (*Nurse−2*)
3.3 Nutritional Supplements	ONS and PN were provided from hospital budget (*Doctor−1 & Nurse−1*)	ONS were initially issued from the hospital then followed up with regular meals (*Nurse−2 & Patient)*
**Theme 4. Patient Outcome**		
4.1 Weight gain	Not achieved as targeted (*Doctor−1 & Nurse−1*)	Achieved (*Nurse−2)*
4.2 Quality of life	Poor, mainly bed bound, Limb weakness. Dependent on basic activities of daily living (*Nurse−1)*	Good. Independent on Basic activities of daily living (*Nurse−2 & Patient*)
4.3 Reconstructive Surgery	Not possible (*Doctor−1 & Nurse −1)*	Successful. 5 months following the initial surgery (*Patient*)
4.4 Final Outcome	Expired, following ICU admission (*Doctor−1 & Nurse−1)*	Discharged home following reconstructive surgery (*Nurse−2 & Patient)*

**Key:** Doctor −1 - Doctor on duty at ward 38 involved with manual CRT.

Doctor −2 - Doctor on duty at surgical professorial unit involved with pump-assisted CRT.

Nurse −1 - Nursing officer on duty at ward 38, involved with manual CRT.

Nurse −2 - Nursing officer on duty at professorial surgical unit with pump-assisted CRT.

Bystander- Manual CRT Patient’s mother

Patient - Patient managed with pump-assisted CRT.

Abbreviations: CRT = chyme reinfusion therapy

PN = parenteral nutrition

ONS = oral nutrition supplements

NG = nasogastric

ALT = alanine aminotransferase

AST = aspartate aminotransferase

## Discussion

Assessing the efficacy of alternative CRT methods is essential to determine which one more effectively improves nutrient absorption and overall nutritional status in SBS patients. Evaluating the cost-effectiveness of manual versus pump-assisted CRT, including equipment costs, healthcare provider time and safety profiles of both techniques helps to understand the risks and benefits associated with each method, including mechanical complications, patient satisfaction and patient outcomes.

### Procedural efficacy

Pump-assisted CRT demonstrated significantly greater procedural efficacy compared to the manual method. The Insides® system reinfused 100 % of the 24 hr output in 3–4 days with minimal manual handling, whereas the manual method reinfused only 40 % of the 24 hr output over > 10 days, with chyme particles being strained out. This disparity was also reflected in the time required for each procedure; the pump-assisted method took only 5 minutes per session, while the manual method took approximately 2 hours. The high frequency and completeness of the automated CRT contributed to a more efficient process and greater nutrient reinfusion.

### Complications and patient experience

Complications were more frequent with the manual method, where the patient experienced abdominal pain and discomfort during and after the procedure. Furthermore, significant challenges for the nursing staff, who had to handle partially formed stools, led to distress and exhaustion. By comparison, there were no significant complications using the pump-assisted method that required manual intervention, providing a more seamless experience for the caregivers and the patient, who reported no discomfort.

### Nutritional and clinical outcomes

The marked differences in nutritional outcomes are partly due to the dissimilar disease pathologies. Consequently, despite slowly increasing the daily energy and protein with NG feeds and sporadic supplementary PN, the patient undergoing manual CRT showed minimal weight gain, poor muscle mass improvement, with fluctuating serum electrolyte levels and micronutrient deficiencies. Targeted albumin and weight gain could not be achieved, and ileostomy reversal was not possible.

In contrast, the patient treated with pump-assisted CRT weaned early from PN to oral nutrition supplements. He gained 3 kg in 8 weeks, showed improved muscle strength, and had better overall nutritional status with favourable albumin, stable hydration and serum electrolyte levels. Ileostomy reversal was achieved in 11 weeks.

### Cost for the patient and resource utilization

From a cost perspective, after the initial pump cost, automated CRT did not necessitate additional nursing staff costs or supplementary equipment that were included in the product package. In contrast, the manual method required expensive supplementary PN due to inadequate chyme reinfusion, extra staff hours and purchase of additional consumables such as wide-bore catheters and strainers, further increasing the cost.

### Patient outcomes and quality of life

Assessing patients’ QOL, including ease of use, comfort, and overall satisfaction is vital, perhaps providing the most critical impact for delivering patient-centered care.

The patient receiving manual CRT experienced gradual deterioration, ultimately resulting in death (for unrelated reasons), without the possibility of reconstructive surgery. On the other hand, the patient managed with pump-assisted CRT showed marked improvements in QOL including increased mobility, independence in daily activities, better appetite and food intake. This patient successfully underwent reconstructive surgery and was discharged after recovery.

### Limitations and future recommendations

The limitation is the small sample size of only two unmatched cases. Moreover, the second patient had a surgical injury to the small bowel, whereas the first patient had both shorter proximal small bowel and diseased residual small bowel, possibly with enteric tuberculosis, that may have contributed to the differences in nutritional outcome. Nevertheless, our study provides a comprehensive comparison between practical aspects of manual and automated CRT, focusing on procedural efficacy, cost, nursing and patient satisfaction.

## Conclusion

Pump-assisted automated chyme reinfusion therapy outperformed the manual method in terms of procedural efficiency, patient satisfaction and cost-effectiveness. It provided a more positive experience for patient and healthcare providers, making it a more viable clinical option. Several key differences between the two methods have been highlighted that can inform future clinical practice and patient care. Our case studies suggest that pump-assisted chyme reinfusion therapy can be considered as the preferred method, particularly for patients with high-output ostomies. Future studies should involve larger cohorts to validate these findings and explore the long-term effects of CRT on intestinal health and surgical outcomes.

## Ethical clearance

Ethics approval was obtained from the local ethical board review committee

## Patients’ consent

A written informed consent was obtained from the key participants. (patient/ward staff)

## Funding

This research did not receive any specific grant from funding agencies in the public, commercial or not-for-profit sectors.

## Declaration of Competing Interest

The authors declare the following financial interests/personal relationships which may be considered as potential competing interests: Gil Hardy reports a relationship with Insides Company Ltd that includes: consulting or advisory. If there are other authors, they declare that they have no known competing financial interests or personal relationships that could have appeared to influence the work reported in this paper.
